# Gastrodin improves preeclampsia‐induced cell apoptosis by regulation of TLR4/NF‐κB in rats

**DOI:** 10.1002/fsn3.1342

**Published:** 2020-01-10

**Authors:** Zhixiong Mei, Baoqin Huang, Xialiu Qian, Yuan Zhang, Benqi Teng

**Affiliations:** ^1^ Department of Obstetrics The Third Affiliated Hospital Sun Yat‐sen University Guangzhou China

**Keywords:** cell apoptosis, gastrodin, MyD88/NF‐κB, PE

## Abstract

To explain gastrodin improved cell apoptosis induced by preeclampsia in vivo and in vitro study. The PE and normal rats were injected with normal saline (Model), low‐dose gastrodin (Gas‐L), medium‐dose gastrodin (Gas‐M), and high‐dose gastrodin (Gas‐H) groups at 50, 100, or 200 mg/kg per day. The rat blood pressure and 24‐hr urine protein level were measured at pregnant days 10, 16, and 20. Evaluating pathology by H&E staining, the cell apoptosis by TUNEL, and MyD88 and NF‐κB (p65) proteins by IHC assay using H/R to simulate PE cell model. Measuring cell proliferation, apoptosis, and MyD88 and NF‐κB (p65) protein expression by MTT, flow cytometry, and WB assay. The SBP, DBP, and 24‐hr urine protein levels were significantly different in PE rats (*p* < .05). The SBP, DBP, and 24‐hr urine protein levels were significantly improved (*p* < .05) in vivo and in vitro. The positive apoptosis cells and apoptosis rate were significantly increased with MyD88 and NF‐κB (p65) proteins upregulation (*p* < .05). The positive apoptosis cells and apoptosis rate were significantly decreased with MyD88 and NF‐κB (p65) proteins depressing in gastrodin‐treated groups with dose‐dependent (*p* < .05). Gastrodin improves PE‐induced cell apoptosis and pathology changed via MyD88/NF‐κB pathway in vitro and in vivo study.

## Graphical abstract

This study showed that gastrodin improves preeclampsia by in vivo study.

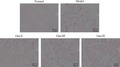

## 1 INTRODUCTION

Preeclampsia (PE) is an idiopathic disorder specific to human pregnancy, which greatly impairs the health of pregnant women and their fetuses despite relatively low incidence. Many maternal, placental, and fetal factors may involve in the occurrence of preeclampsia. Oxidative stress and inflammatory response have been widely concerned as important potential factors for preeclampsia in recent years, and inhibition of oxidative stress and inflammatory response to reduce the incidence of preeclampsia is a current research focus in this field (Alijotas‐Reig, Esteve‐Valverde, & Ferrer‐Oliveras, 2017; Gathiram & Moodley, 2016; Phipps, Prasanna, & Brima, 2016). Many studies have confirmed that the normal development of the placenta depends on the precise temporal and spatial regulation by apoptosis and its normal function rests on the balance between the proliferation, differentiation, and apoptosis of trophoblasts. Abnormal placental function is closely related to the abnormal apoptosis of the placenta (Shahid, Khalid, & Fatima, 2019; Travaglino et al., 2019).

Gastrodin (gas) is the main bioactive component of Gastrodia elata and exhibits anti‐inflammatory, anti‐oxidation, anti‐tumor, and brain protection activities (Du et al., 2016; Qin et al., 2018; Wang et al., 2014). Studies have confirmed that overexpression of inflammatory factors is the major reason for inducing preeclampsia (Albrecht et al., 2019; Kaminski, Ellwanger, & Sandrim, 2019). On this basis, it was speculated that gas may improve the occurrence of preeclampsia via inhibiting the inflammatory response.

### 1.1 Laboratory animal grouping

Forty‐eight healthy CL pregnant Wistar rats weighing 200–250 g were provided by the Experimental Animal Center of Nanjing Medical University. Animals were maintained at room temperature of 7–27°C with a relative humidity of 50%–70%. The first gestation day (GD 1) was determined as the day when a vaginal plug was present with microscopic observation of sperms in the vaginal of the experimental mice. The present study was reviewed and approved by the Ethics Committee of the Third Affiliated Hospital.

Rats were divided into two groups according to the random number table, with nine in the control group and 36 in the preeclampsia group. Animals were maintained till GD 12.

## 2 METHODS

### 2.1 Establishment of an animal model of preeclampsia

On GD 13, pregnant rats in the preeclampsia group were subcutaneously injected with N‐nitro‐L‐arginine methyl ester (L‐NAME) (Sigma, USA; batch number: 20120925) at a dose of 100 mg/(kg d) according to the method previously described ( Pellicer, Herraiz, & Leal, 2011) to establish a preeclampsia animal model. Animals in the control group were injected subcutaneously with normal saline at 1.5 ml/d. Corresponding injections were given till GD 21. This study was approved by Ethics Committee of the Third Affiliated Hospital (No. 2018031003).

### 2.2 Gas intervention

Rats in the preeclampsia group were randomly divided into four subgroups (*n* = 9 per subgroup) on the 4th day of modeling (GD 16). Animals in the blank intervention group were given intraperitoneal injection of normal saline at 1.5 ml/d and those in the three intervention subgroups, including the low‐ (Gas‐L), medium‐ (Gas‐M), and high‐dose gas intervention subgroup (Gas‐H), were given intraperitoneal injection of gas (Kunming Pharmaceutical Group Co., Ltd., purity >98%) at 50, 100, and 200 mg/(kg d), respectively, according to the method previously described (Guo et al., 2014; Lin et al., 2014). All rats were injected till GD 21 when animals were killed.

### 2.3 Cell culture and modeling

HTR/SVneo cells (Jiangsu Kaiji Biotechnology Co. Ltd.) were inoculated in RPMI1640 medium containing 10% fetal bovine serum in a constant temperature CO_2_ incubator at 37℃. The culture medium was changed for every 3 days. The cells were digested using 0.25% pancreatin at the logarithmic growth stage. The oxygen concentration of constant temperature three‐gas incubator at 37°C was set as 1%. HTR/SVneo cells were inoculated into a 6‐well plate with 1 × 10^6^/well. After the cells adhered to the wall, the cells in each group were correspondingly treated and cultured in the three‐gas incubator under an anoxic environment for 48 hr.

HTR/SVneo cells of the normal group were cultured in a normal environment. HTR/SVneo cells of the model group and all gas groups were cultured in an anoxic environment. The cells in the model group, gas‐L, gas‐M, and gas‐H groups were pretreated with normal medium containing 50, 100, and 200 mg/ml gastrodin, respectively.

### 2.4 Blood pressure measurement in rats

The caudal arterial pressure was measured by noninvasive blood pressure monitoring system (ALC‐NIBP system; Shanghai ALCBio) for each rat on GDs 10, 16, and 20 according to the procedure previously described (Fernández Celadilla, Carbajo Rueda, & Muñoz, 2005).

### 2.5 24‐hr urine protein test in rats

Twenty‐four‐hour urine samples were collected on GDs 10, 16, and 20, respectively (rats were housed in 41 700/1/2/3 metabolic cages manufactured in Italy for collecting urine, and diet for each group was well‐balanced during the feeding period), and 24‐hr urine protein concentration was determined using the Beckman Coulter AU5831 automatic biochemical analyzer.

### 2.6 Immunohistochemistry

The placenta tissue was embedded in paraffin using the procedure previously described (Kasture, Sundrani, & Joshi, 2018), and the expression of MyD88 and NF‐κB in placenta trophoblasts was observed by immunohistochemical DAB staining. The SCANSCOPE medical image analysis system was used to determine the gray value, which was inversely proportional to the degree of protein expression, that is, lower gray value indicated higher expression.

### 2.7 Histological changes of placenta

H&E staining was used followed by light microscopic observation (200×) to determine the histological changes of the placenta.

### 2.8 Syncytiotrophoblast apoptosis assay

Assay was conducted according to the instructions provided with the kit. Cell apoptosis was microscopically examined under a 400× microscope with 20 fields for each specimen. TUNEL‐positive cells (colored brown‐yellow or brown) and total cells were counted, and the apoptosis rate of syncytiotrophoblasts was calculated.

### 2.9 MTT assay

The proliferation activity of cells in each group was measured by MTT assay. The cells were inoculated into a 96‐well plate with 1.0 × 10^5^ cells/well. The cells in each group were treated in different ways and continuously cultured for 48 hr. Then, 20 μL/well MTT was added for continuous culture. Four hours later, the upper solution was discarded, and 1,300 μL/well DMSO was added and dissolved by shaking for 12 min. The optical density (OD) value at 470 nm was measured using enzyme‐labeling instrument.

### 2.10 Detection of apoptosis by flow cytometry

The cells were inoculated into a 96‐well plate with 1.0 × 10^5^ cells/well. Cells in each group were treated in different ways and continuously cultured for 48 hr. The cells were collected, washed with appropriate PBS for three times, and stored in 75% precooled ethanol overnight at 4°C. After washing with PBS three times, the cells were incubated in 20 μL RNase at 37°C for 20 min, and then, stained with 20 μL propidium iodide (PI) at dark and incubated at 4°C for 30 min. The cells were analyzed using a FACScan flow cytometer, and the apoptotic cells were counted by the ModFit LT program for cell cycle analysis.

### 2.11 Western blotting

The cells in each group were lysed using cell lysate (containing PMSF). After centrifugation at 8,000 *g* for 15 min, the supernatant was collected and placed at 4°C for further use. Protein concentration was detected using BCA detection kit according to the instructions. Semiquantitative analysis was carried out according to the detected protein concentration. The protein was separated using 10% SDSPAGE, transmembraned with the whole‐wet method at 350 Ma for 90 min, sealed in 5% skimmed milk at room temperature for 2 hr, and washed with PBS five times (3 min/time). The primary antibodies, MyD88 and NF ‐κB monoclonal antibodies (1:1,000 dilution) or GAPDH monoclonal antibody (1:1,000 dilution), were added and incubated at room temperature for 2.5 hr, followed by membrane washing with PBST three times and PBS twice (5 min/time). At room temperature, fluorescence‐labeled secondary antibody (IRDye 680RD DonkeyAnti‐mouse IgG, 1:20,000 dilution) was added and incubated for 1.5 hr, followed by membrane washing with PBST three times, PBS twice, and high‐pressure ddH_2_O twice (3 min/time). The relative protein expression was analyzed using the Odyssey^®^ infrared imaging system.

### 2.12 RT‐qPCR assay

The HTR/SVneo cell of different groups or 100 mg placental tissue was taken, and 1‐ml Trizol lysis buffer was added. Total RNA was extracted according to the instructions for Trizol reagents and reverse‐transcribed into cDNAs, followed by real‐time PCR. PCR cycling conditions included predenaturation at 95°C for 15 min, 40 cycles of denaturation at 95°C for 30 s, annealing at 60°C for 60 s, and extension at 60°C for 60 s. Primer sequences included F: 5′‐GGATCCATGGCTGCAGGAGGTCC‐3′ and R: 5′‐GGGCCCGGGCAGGGACAAGGCCTTGG‐3′ for MyD88; F: 5′‐GAGAGCCCTTGCATCCTTTA‐3′ and R: 5′‐CTTCCCTTTGGTCTTTCTGT‐3′ for NF‐κB; and F: 5′‐CATCTTCCAGGAGCGAGACC‐3′ and R: 5′‐CTCGTGGTFCACACCCATCA‐3′ for GAPDH. The relative expression level was calculated by 2^−ΔΔCT^ method using GAPDH as an internal reference.

### 2.13 Cell immunofluorescence

After cells in each group were correspondingly processed for 48 hr, we let the specimens (cell smears) naturally dry. Then, they were immersed in 4% paraformaldehyde fixation for 30 min or overnight for the purpose of improving permeability of cells. Then, the cells were subjected to immersion cleaning thrice; each time, the immersion cleaning should last for 3 min. Moreover, two drops of 3% H_2_O_2_‐methanol solution were added on each slide that was sealed at room temperature of 15–25℃ for 10 min, rinsed in PBS for 3 min. 50‐100 μl ready‐to‐use goat serum was added dropwise to incubate the specimens at room temperature for 20 min; then, p‐NF‐κ B (p65) (Abcam, ab16502, UK) (1:100) primary antibody was added dropwise for 2‐hr incubation in wet box. After they were immersed in PBS and rinsed for three times, 100 μl FITC second antibody was added dropwise for 1‐hr incubation at 37°C in a dark place, following which, the specimen should be subjected to immersion cleaning three times in PBS. On each slide, the prepared DAPI staining fluid of 50–100 μl was added in a dropwise manner and then the slide was placed at room temperature for 5 min in a dark place. Afterward, the slide should be sealed with antiquenching mounting gel and then put under a microscope to observe protein expressions in cells. During observation, pictures of 3 sites of overexpression were taken and preserved.

### 2.14 Statistical analysis

Statistical analysis was performed using SPSS 19.0 software. Measured data with normal distribution were presented as mean ± *SD*. Two independent samples were compared by *t* test. For comparison among groups, *F* test was applied for multiple groups and LSD‐*t* test for pairwise comparison. *p* < .05 was considered statistically significant using a significance level of *α* = 0.05.

## 3 RESULTS

Comparison of caudal arterial pressure and urine protein level between the control group and the preeclampsia group on GD 10 (before modeling).

No significant differences in systolic blood pressure (SBP), diastolic blood pressure (DBP), and 24‐hr urine protein were observed between the two groups on GD 10 (all *p* > .05) (Table 1).

**Table 1:** Comparison of caudal arterial pressure and urine protein level between the control group and the preeclampsia group on GD 10 (before modeling) (mean ± *SD*)

Group	*n*	SBP (mmHg)	DBP (mmHg)	24‐hr Urine protein (mg)
Normal	9	110.54 ± 6.01	81.25 ± 7.98	6.34 ± 0.84
Model	36	109.87 ± 5.99	79.68 ± 7.56	6.48 ± 0.59
*t*		0.98	0.67	1.35
*p*		>.05	>.05	>.05

Note

1 mmHg = 0.133 kPa

Comparison of caudal arterial pressure and urine protein level between the control group and each preeclampsia subgroup on GD 16 (4 days after modeling, no gas treatment).

Compared with the control group, SBP, DBP, and 24‐hr urinary protein levels were significantly increased in all preeclampsia subgroups (all *p* < .01), and no statistically significant differences in these values were noted among preeclampsia subgroups (all *p* > .05) (Table 2).

**Table 2:** Comparison of caudal arterial pressure and urine protein level between the control group and each preeclampsia subgroup on GD 16 (4 days after modeling, no gas treatment) (mean ± *SD*)

Group	*n*	SBP (mmHg)	DBP (mmHg)	24‐hr Urine protein (mg)
Normal	9	110.58 ± 4.54	80.18 ± 7.61	6.41 ± 0.61
Model	9	129.57 ± 5.12***	89.51 ± 6.54***	7.81 ± 0.49***
Gas‐L	9	130.16 ± 4.99***	89.41 ± 5.16***	7.79 ± 0.51***
Gas‐M	9	130.54 ± 5.29***	89.61 ± 5.88***	7.77 ± 0.48***
Gas‐H	9	129.84 ± 5.97***	90.13 ± 6.54***	7.61 ± 0.53***
*F*		66.97	52.11	46.59
*p*		<.001	<.001	<.001

*** *p* < .001, compared with Normal group.

Comparison of caudal arterial pressure and urine protein level between the control group and each preeclampsia subgroup on GD 20 (8 days after modeling and 4 days of gas treatment).

Systolic blood pressure, DBP, and 24‐hr urinary protein levels in the model group as well as in the Gas‐L, Gas‐M, and Gas‐H subgroups were significantly higher than those in the control group (all *p* < .05) and these corresponding values in the model group were significantly higher than those in the Gas‐L, Gas‐M, and Gas‐H subgroups (all *p* < .05). There were no significant differences in the SBP, DBP, and 24‐hr urinary protein levels among the Gas‐L, Gas‐M, and Gas‐H subgroups (*p* > .05) (Table 3).

**Table 3:** Comparison of caudal arterial pressure and urine protein level between the control group and each preeclampsia subgroup on GD 20 (8 days after modeling, and 4 days of gas treatment) (mean ± *SD*)

Group	*n*	SBP (mmHg)	DBP (mmHg)	24‐hr Urine protein (mg)
Normal	9	110.49 ± 3.54	79.59 ± 6.54	6.41 ± 0.64
Model	9	137.59 ± 5.51***	90.15 ± 5.10***	7.85 ± 0.44***
Gas‐L	9	131.44 ± 4.89#	88.06 ± 4.54#	6.03 ± 0.51#
Gas‐M	9	122.74 ± 5.14## ^,^ &	85.11 ± 4.82## ^,^ &	5.55 ± 0.41## ^,^ &
Gas‐H	9	112.06 ± 4.54### ^,^ && ^,^ $	80.24 ± 5.41### ^,^ && ^,^ $	4.95 ± 0.43### ^,^ && ^,^ $
*F*		85.64	35.77	50.12
*p*		<.001	<.001	<.001

*** *p* < .001, compared with Normal group; ^#^ *p* < .05, ^##^ *p* < .01, ^###^ *p* < .001, compared with Model group; ^&^ *p* < .05, ^&&^ *p* < .01, compared with gas‐L group; ^$^ *p* < .05 compared with gas‐M.

### 3.1 Comparison of histological changes of placenta among groups

**Figure 1:**
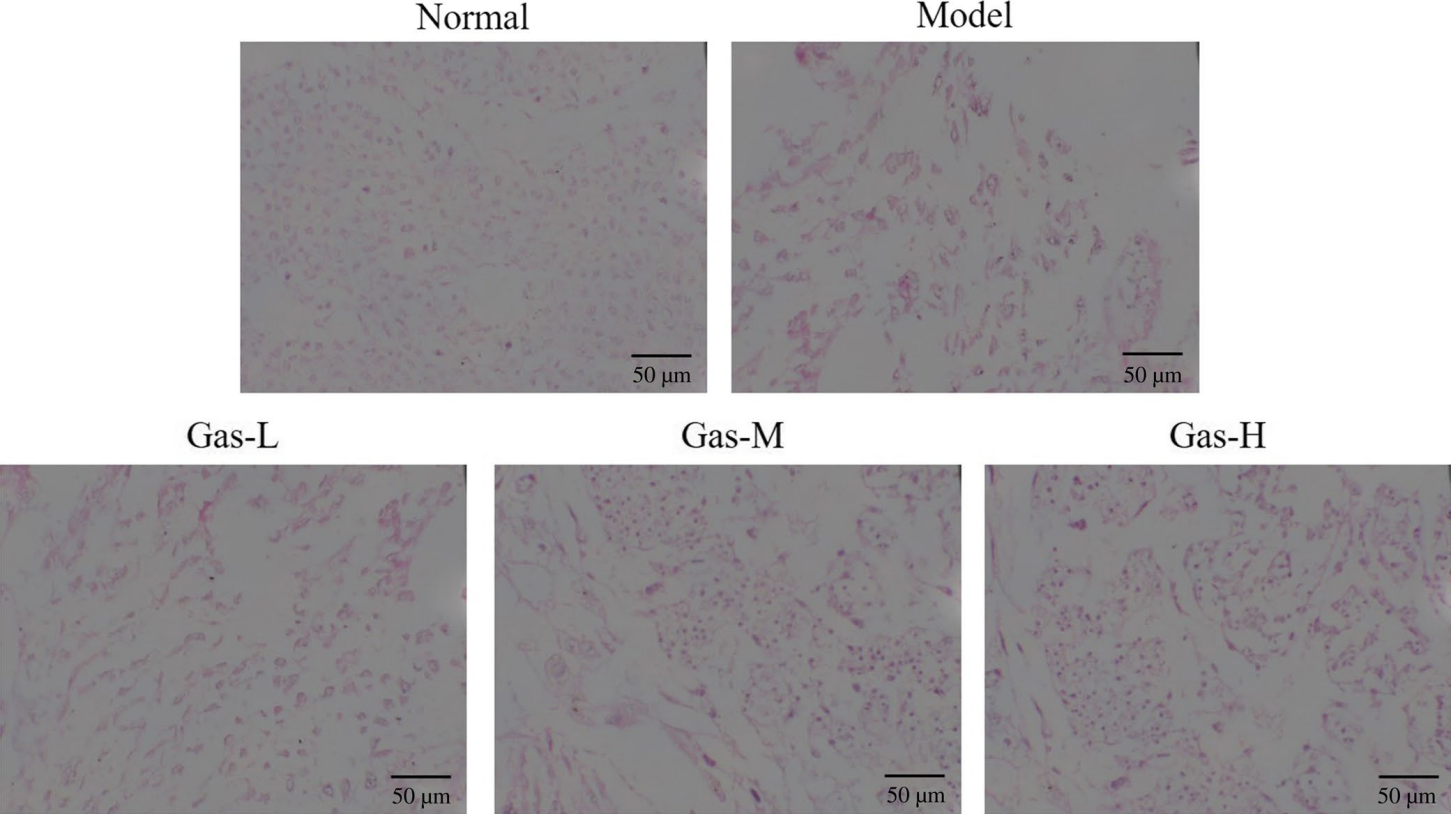
Histological of different groups by H & E staining (200×). Normal: Normal control group; Model: The PE model group was treated with NS; Gas‐L: The PE model rats were treated with 50 mg/kg gastrodin; Gas‐M: The PE model rats were treated with 100 mg/kg gastrodin; Gas‐H: The PE model rats were treated with 200 mg/kg gastrodin

For animals in the normal group, the structure of villus was clear in the placenta and the fetal blood vessels in the villi expanded to the syncytiotrophoblast layer with maternal sinusoids normal in size and rich in blood. While in the model group, fetal vascular red blood cells accumulated in the main villi, resulting blood stasis; the structure of small villus was blurred and ischemic‐like changes occurred in fetal blood vessels which failed to expand to the syncytiotrophoblast layer, and maternal sinusoidal dilatation was observed with reduced amount of blood. The above pathological changes in the placenta were significantly improved in rats of the Gas‐L, Gas‐M, and Gas‐H subgroups (Figure 1).

### 3.2 Comparison of apoptosis and expression of related genes in syncytiotrophoblasts among groups

**Figure 2:**
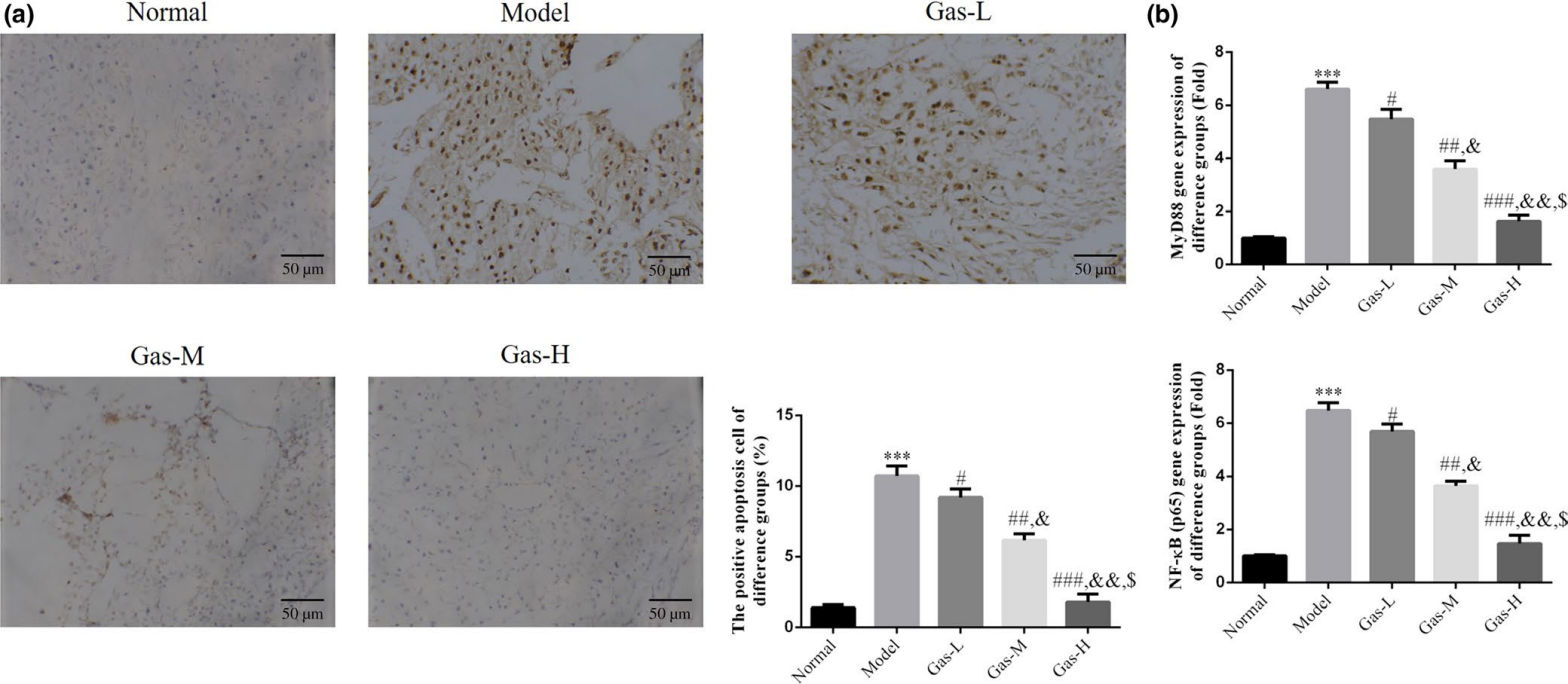
The positive apoptosis cell number by TUNEL assay and relative mRNA expression by RT‐qPCR. (a) The positive apoptosis cell number by TUNEL assay (200×). Normal: Normal control group; Model: The PE model group was treated with NS; Gas‐L: The PE model rats were treated with 50 mg/kg gastrodin; Gas‐M: The PE model rats were treated with 100 mg/kg gastrodin; Gas‐H: The PE model rats were treated with 200 mg/kg gastrodin. ***p < .001, compared with Normal group; #*p* < .05, ##*p* < .01, ###*P* < .001, compared with Model group; &*p* < .05, &&*p* < .01, compared with Gas‐L group; $*p* < .05 compared with gas‐M. (b) The relative mRNA expressions of different groups by RT‐qPCR assay. Normal: Normal control group; Model: The PE model group was treated with NS; Gas‐L: The PE model rats were treated with 50 mg/kg gastrodin; Gas‐M: The PE model rats were treated with 100 mg/kg gastrodin; Gas‐H: The PE model rats were treated with 200 mg/kg gastrodin. ***p < .001, compared with Normal group; #*p* < .05, ##*p* < .01, ###*p* < .001, compared with Model group; &*p* < .05, &&P < .01, compared with gas‐L group; $*p* < .05 compared with gas‐M

Comparing with apoptosis rate of the normal group, apoptosis rate of the placenta syncytiotrophoblasts of model group was significantly increased (*p* < .001). After gas intervention, the apoptosis rates of the three gas intervention subgroups were significantly inhibited compared with that of the model group (*p* < .05, *p* < .01, and *p* < .001, respectively). At the same time, significant difference in apoptosis rate was observed among these gas intervention subgroups (*p* < .05, Figure 2a). MyD88 and NF‐κB (p65) genes levels were detected by RT‐qPCR. Compared with the normal group, the expression levels of MyD88 and NF‐κB (p65) in the model group were significantly increased (*p* < .001, respectively, Figure 2b). After gas intervention, the expression levels of MyD88 and NF‐κB (p65) in the three gas intervention subgroups were significantly inhibited compared with that of the model group (*p* < .05, *p* < .01, and *p* < .001, respectively, Figure 2b). Significant differences in the expression levels of MyD88 and NF‐κB (p65) were noted among these gas intervention subgroups (*p* < .05 and *p* < .01, respectively, Figure 2b).

### 3.3 Expression levels of placental MyD88 and NF‐κB (p65) proteins in each group

**Figure 3:**
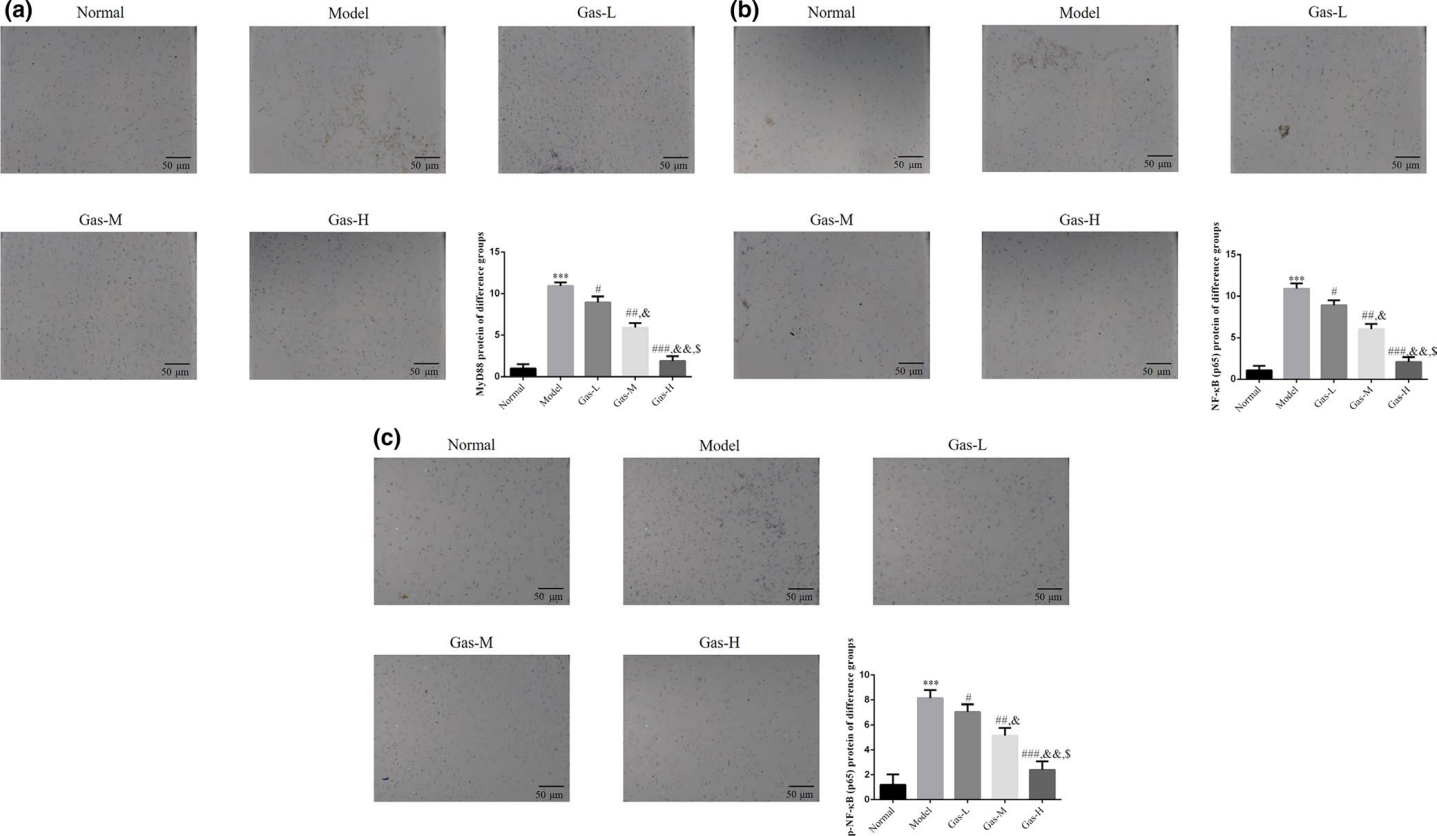
The relative proteins expressions by IHC assay. (a) The MyD88 protein expression of different groups by IHC assay (200×). Normal: Normal control group; Model: The PE model group was treated with NS; Gas‐L: The PE model rats were treated with 50 mg/kg gastrodin; Gas‐M: The PE model rats were treated with 100 mg/kg gastrodin; Gas‐H: The PE model rats were treated with 200 mg/kg gastrodin. ***p < .001, compared with Normal group; #*p* < .05, ##*p* < .01, ###*p* < .001, compared with Model group; &*p* < .05, &&*p* < .01, compared with gas‐L group; $*p* < .05 compared with gas‐M. (b) The NF‐κB (p65) protein expression of different groups by IHC assay (200×). Normal: Normal control group; Model: The PE model group was treated with NS; Gas‐L: The PE model rats were treated with 50 mg/kg gastrodin; Gas‐M: The PE model rats were treated with 100 mg/kg gastrodin; Gas‐H: The PE model rats were treated with 200 mg/kg gastrodin. ***p < .001, compared with Normal group; #*p* < .05, ##*p* < .01, ###*p* < .001, compared with Model group; &*p* < .05, &&*p* < .01, compared with gas‐L group; $*p* < .05 compared with gas‐M. (c) The p‐NF‐κB (p65) protein expression of different groups by IHC assay (200×). Normal: Normal control group; Model: The PE model group was treated with NS; Gas‐L: The PE model rats were treated with 50 mg/kg gastrodin; Gas‐M: The PE model rats were treated with 100 mg/kg gastrodin; Gas‐H: The PE model rats were treated with 200 mg/kg gastrodin. ***p < .001, compared with Normal group; #*p* < .05, ##*p* < .01, ###*p* < .001, compared with Model group; &*p* < .05, &&*p* < .01, compared with gas‐L group; $*p* < .05 compared with gas‐M

MyD88, NF‐κB (p65), and p‐NF‐κB (p65) protein levels were detected by IHC. The expression levels of these two proteins in the model group were significantly increased compared with those of the normal group (*p* < .001, respectively, Figure 3a‐c). After gas intervention, the expression levels of MyD88, NF‐κB (p65), and p‐NF‐κB (p65)proteins in the three gas intervention subgroups were significantly inhibited compared with those of the model group (*p* < .05, *p* < .01, or *p* < .001, respectively, Figure 3a‐c). Meanwhile, there were significant differences in the expression levels of MyD88, NF‐κB (p65), and p‐NF‐κB (p65) among the gas intervention subgroups (*p* < .05 or *p* < .01, Figure 3a‐c).

### 3.4 Gastrodin improves HTR/SVneo cell apoptosis induced by H/R environment

**Figure 4:**
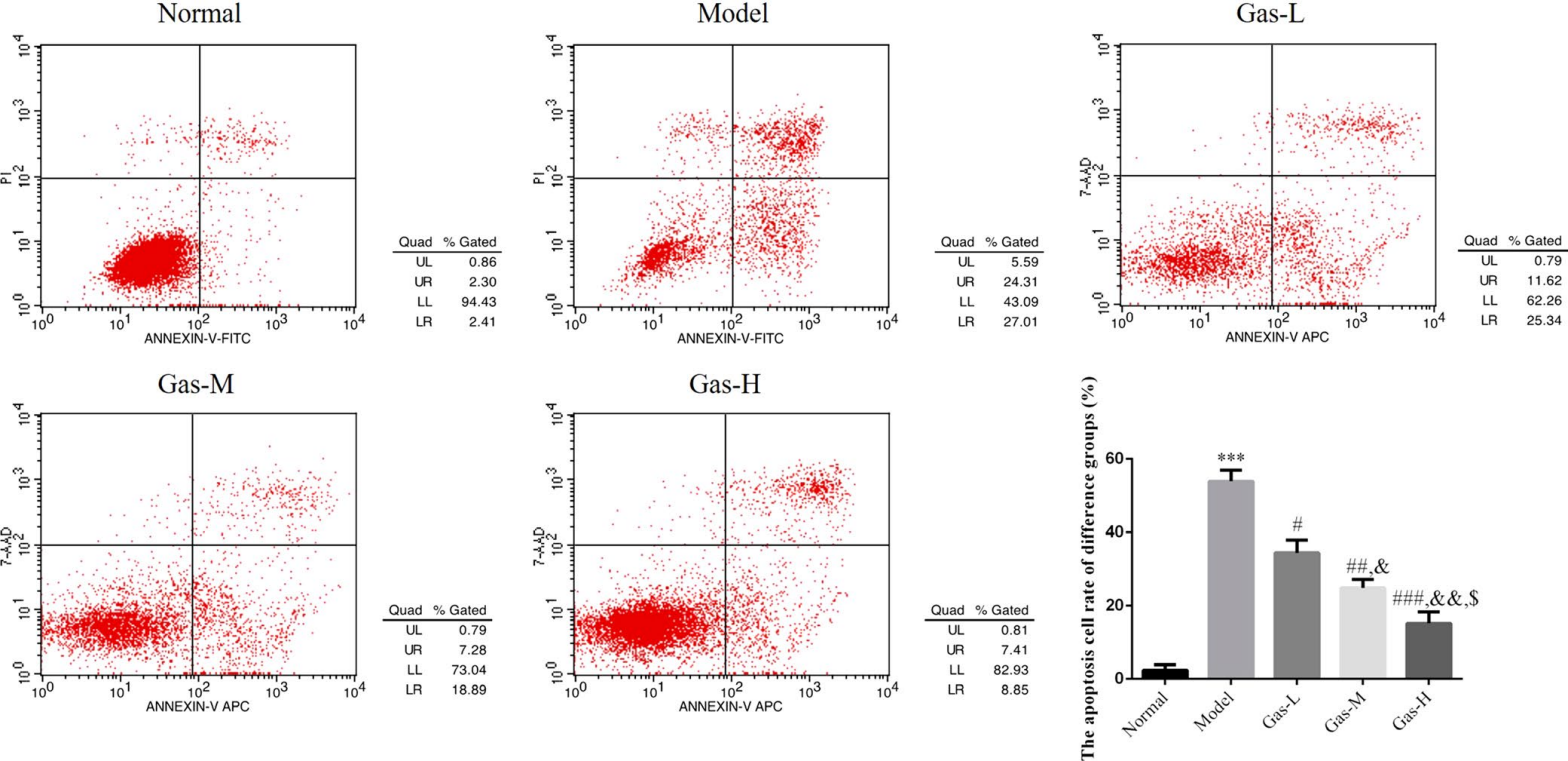
The cell apoptosis rate of different groups by flow cytometry. Normal: Normal control group; Model: The HTR/SVneo cells were treated with hypoxia; Gas‐L: The HTR/SVneo cells were treated with 50 mg/ml gastrodin hypoxia; Gas‐M: The HTR/SVneo cells were treated with 100 mg/ml gastrodin hypoxia; Gas‐H: The HTR/SVneo cells were treated with 200 mg/ml gastrodin hypoxia. ***p < .001, compared with Normal group; #*p* < .05, ##*p* < .01, ###*p* < .001, compared with Model group; &*p* < .05, &&*p* < .01, compared with gas‐L group; $*p* < .05 compared with gas‐M

With H/R environment‐treated, the HTR/SVneo cell apoptosis rate was significantly increased compared with normal group by flow cytometry (*p* < .001, Figure 4); however, the HTR/SVneo cell apoptosis rates were significantly depressed compared with that of model group (*p* < .05, respectively, Figure 4) with gastrodin‐treated. Meanwhile, there were dose‐dependent in gastrodin treatment.

### 3.5 Gastrodin had effects on relative protein and gene expressions by WB and RT‐qPCR assay

**Figure 5:**
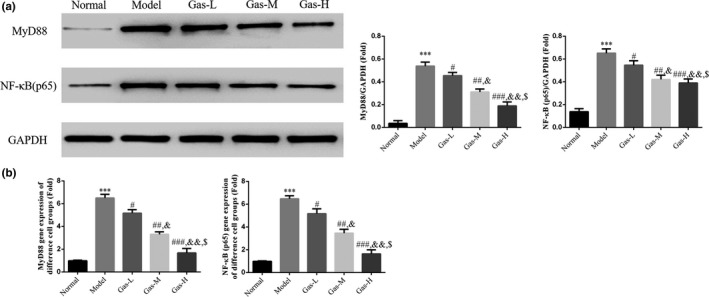
The relative mRNA and protein expressions by RT‐qPCR and WB assay. (a) The relative protein expressions of different groups by WB assay. Normal: Normal control group; Model: The HTR/SVneo cells were treated with hypoxia; Gas‐L: The HTR/SVneo cells were treated with 50 mg/ml gastrodin hypoxia; Gas‐M: The HTR/SVneo cells were treated with 100 mg/ml gastrodin hypoxia; Gas‐H: The HTR/SVneo cells were treated with 200 mg/ml gastrodin hypoxia. ***p < .001, compared with Normal group; #*p* < .05, ##*p* < .01, ###*p* < .001, compared with Model group; &*p* < .05, &&*p* < .01, compared with gas‐L group; $*p* < .05 compared with gas‐M. (b) The relative mRNA expression of different groups by RT‐qPCR assay. Normal: Normal control group; Model: The HTR/SVneo cells were treated with hypoxia; Gas‐L: The HTR/SVneo cells were treated with 50 mg/ml gastrodin hypoxia; Gas‐M: The HTR/SVneo cells were treated with 100 mg/ml gastrodin hypoxia; Gas‐H: The HTR/SVneo cells were treated with 200 mg/ml gastrodin hypoxia. ***p < .001, compared with Normal group; #*p* < .05, ##*p* < .01, ###*p* < .001, compared with Model group; &*p* < .05, &&*p* < .01, compared with Gas‐L group; $*p* < .05 compared with Gas‐M

By WB assay, the MyD88 and NF‐κB (p65) protein expressions of model group were significantly increased compared with those of NC group (*p* < .001, respectively, Figure 5a); with gastrodin supplement, the MyD88 and NF‐κB (p65) protein expressions of gastrodin‐treated groups were significantly depressed compared with those of model group (*p* < .05, respectively, Figure 5a). By RT‐qPCR assay, the MyD88 and NF‐κB (p65) mRNA expression of Model group was significantly increased compared with those of NC group (*p* < .001, respectively, Figure 5b); with gastrodin supplement, the MyD88 and NF‐κB (p65) mRNA expression of gastrodin‐treated groups was significantly depressed compared with those of model group (*p* < .05, respectively, Figure 5b). There were dose‐dependent in gastrodin‐treated groups in MyD88 and NF‐κB (p65) proteins and gene expression.

### 3.6 Gastrodin had effects to p‐NF‐κB (p65) protein expression in different cell groups

**Figure 6:**
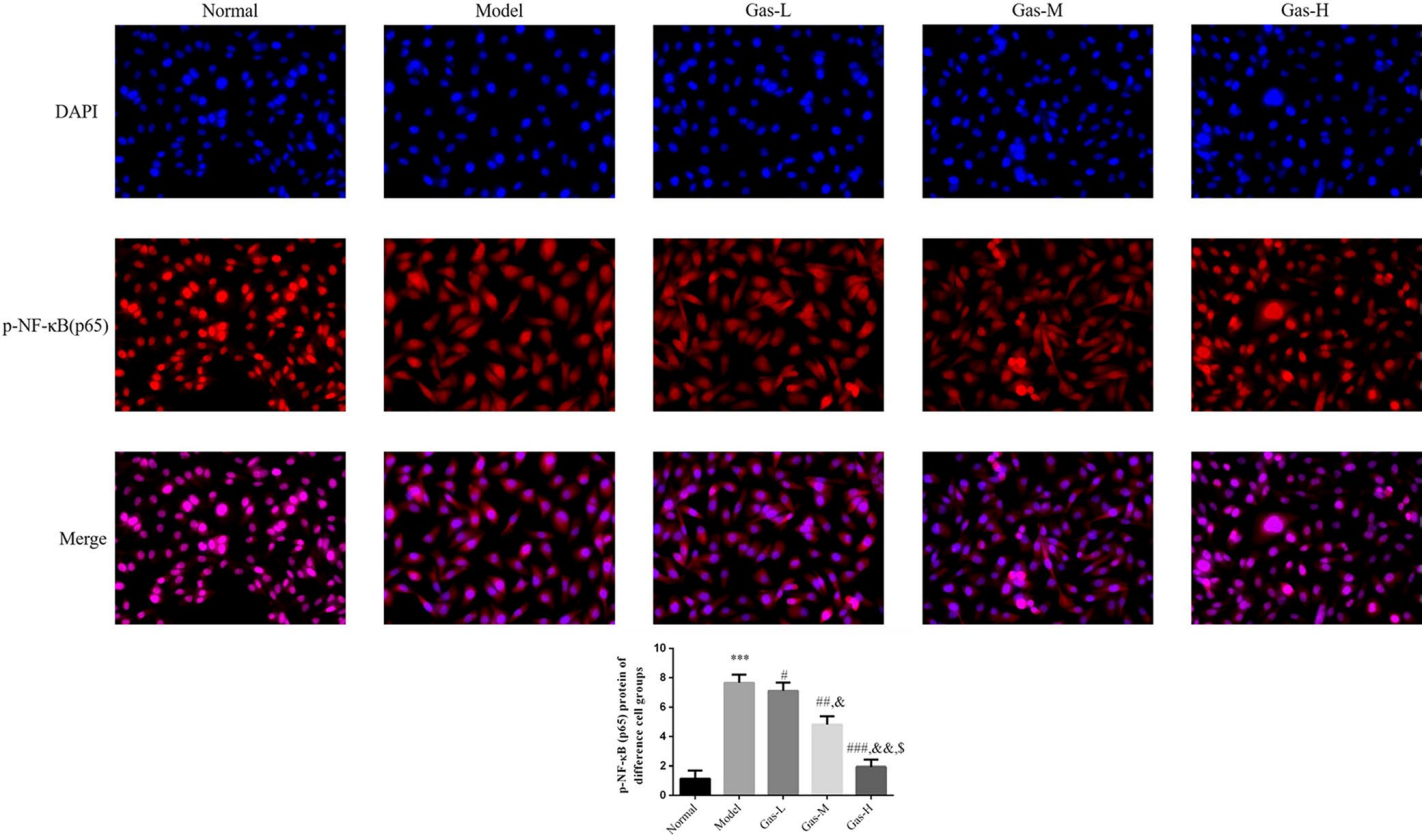
p‐NF‐κB (p65) protein expression in different cell groups by cellular immunofluorescence. Normal: Normal control group; Model: The HTR/SVneo cells were treated with hypoxia; Gas‐L: The HTR/SVneo cells were treated with 50 mg/ml gastrodin hypoxia; Gas‐M: The HTR/SVneo cells were treated with 100 mg/ml gastrodin hypoxia; Gas‐H: The HTR/SVneo cells were treated with 200 mg/ml gastrodin hypoxia. ***p < .001, compared with Normal group; #*p* < .05, ##*p* < .01, ###*p* < .001, compared with Model group; &*p* < .05, &&*p* < .01, compared with gas‐L group; $*p* < .05 compared with gas‐M

By cellular immunofluorescence, the p‐NF‐κB (p65) was expressed in cytoplasm, the p‐NF‐κB (p65) protein expressions of Model group were significantly increased compared with those of NC group (*p* < .001, respectively, Figure 6); with gastrodin supplement, the p‐NF‐κB (p65) protein expressions of gastrodin‐treated groups were significantly depressed compared with those of model group (*p* < .05, respectively, Figure 6).

## 4 DISCUSSION

Gastrodia elata, a precious treasure in traditional Chinese medicine, has been proven to be effective in the treatment of various diseases with a favorable safety profile. Gastrodin is the bioactive component of Gastrodia elata and shows high activities in sedation, blood supply improvement, anti‐inflammation, blood vessel function regulation, and neuroprotection (Liu et al., 2018; Wang, Wang, & Duan, 2018). Studies have demonstrated that placental trophoblasts in patients with preeclampsia undergo excessive apoptosis and the degree of apoptosis is related to the severity of the disease (Heazell, Buttle, & Baker, 2008). It is currently believed that various pathogenic factors including genetic background may increase the apoptosis of trophoblasts, block the process of revascularization of the distal uterine spiral artery, and induce insufficient blood supply to the placenta and consequently further ischemia and hypoxia, leading to the occurrence and development of preeclampsia (Fisher, (2015)). However, the specific mechanism of placental trophoblast apoptosis in preeclampsia patients is not fully elucidated and need for effective clinical intervention remains unmet although a large number of studies have been done in recent years. Zhang et al (Zhang et al., 2018) found that cell apoptosis can be inhibited by downregulating the expression of NF‐κB mRNA and protein. The anti‐apoptotic effect of Gas may provide new ideas for the inhibition of apoptosis in preeclampsia.

Increased caudal arterial pressure and 24‐hr urinary protein level are important indicators for the successful establishment of preeclampsia animal model and the levels of these indicators increase with the prolongation of drug intervention, making them a useful animal model for drug intervention studies (Kaya et al., 2011). The results of the present study showed that the caudal arterial pressure and the urine protein further increased over the L‐NAME treatment time in the preeclampsia group and the SBP, DBP, and 24‐hr urinary protein levels were significantly reduced after intervention with low, medium, and high dose of Gas compared with those of the blank intervention group, suggesting that short‐term drug intervention exerted therapeutic effect although the corresponding indicators were still higher than that of the control group.

Cell death takes two distinct models, necrosis and apoptosis. Necrosis is a degenerative process involving cell swelling and the release of intracellular substances after cell membrane rupture, leading to inflammatory response, while apoptosis is not associated with inflammation. Formigli L et al. recently proposed a cell death model between apoptosis and necrosis, which is called “aponecrosis.” He pointed out that “when syncytial cells undergo excessive apoptosis with inadequate time available for the temporally and spatially regulated apoptosis program to execute complete apoptotic procedure before the cells detach, necrotic release accompanied with initial apoptosis occurs (Formigli et al., 2000). This necrotic release may cause local placental lesions as well as systemic inflammatory responses.” At the same time, in a normal pregnancy, macrophages inhibit the secretion of pro‐inflammatory factors such as TNF‐α and IFN‐γ and promote the secretion of anti‐inflammatory factors and immunosuppressive substances. However, the presence of excessive apoptosis beyond the clearance ability of phagocytic cells disrupts the anti‐inflammatory response, and the increased pro‐inflammatory factors promote the death of trophoblasts (Huppertz, Kadyrov, and Kingdom (2006)). When inflammatory factors increase, the secretion of vasoactive diastolic factors such as NO and prostacyclin decreases and the vasoconstrictor factor thromboxane A2 increases, leading to the occurrence of preeclampsia (Perucci, Corrêa, and Dusse (2017)). Related studies have shown that activation of the MyD88/NF‐κB signaling pathway contributes to inflammatory response (Wang, Dong, & Wang, 2019; Zhang & Zeng, 2019) and cell apoptosis (Li et al., 2015; Zheng, Shen, & Ye, 2019). The results of the present study showed that preeclampsia‐induced apoptosis of placental cells was effectively inhibited after Gas treatment, and the underlying mechanism may involve downregulated activity of MyD88/NF‐κB signaling pathway.

In summary, gas effectively improves inflammation‐induced placental cell apoptosis and related pathology by inhibiting the activation of the MyD88/NF‐κB signaling pathway.

## 5 FUTURE PERSPECTIVES

In our study, we just researched gas played the treatment of PE in cell apoptosis; however, cell apoptosis is just one result which lead PE development, the cell invasion and migration abilities damaged are another result. In our future study, we will research gas improve PE development in cell invasion and migration.

## CONFLICT OF INTEREST

None.

## ETHICAL APPROVAL

This study was approved by Ethics Committee of the Third Affiliated Hospital of Sun Yat‐sen University.
